# The Role of Dipeptidyl Peptidase Inhibitors in Pulmonary Diseases

**DOI:** 10.3390/biomedicines14051008

**Published:** 2026-04-28

**Authors:** Theodoros Panou, Paschalis Steiropoulos, Fotios Drakopanagiotakis

**Affiliations:** Department of Respiratory Medicine, Medical School, Democritus University of Thrace, 68100 Alexandroupolis, Greecesteiropoulos@yahoo.com (P.S.)

**Keywords:** lung, respiratory, dipeptidyl peptidase, dipeptidyl peptidase-1, cathepsin C, dipeptidyl peptidase-4

## Abstract

The dipeptidyl peptidase (DPP) family comprises enzymes with important metabolic and immunomodulatory properties. This narrative review summarizes recent clinical and experimental evidence on the role of DPP-1, DPP-4, DPP-9, and DPP-10 in pulmonary diseases. The strongest translational evidence currently supports DPP-1 inhibition in non-cystic fibrosis bronchiectasis, where brensocatib reduces exacerbations and prolongs time to first exacerbation, with additional DPP-1 inhibitors in development. By contrast, the roles of DPP-4, DPP-9, and DPP-10 are supported mainly by preclinical studies in pulmonary hypertension, acute lung injury (ALI)/acute respiratory distress syndrome (ARDS), pulmonary fibrosis, asthma, non-small cell lung cancer (NSCLC), and nonsteroidal anti-inflammatory drugs (NSAIDs)/aspirin-exacerbated respiratory disease. Across these models, DPP inhibition modulates inflammation, protease activation, epithelial- or endothelial-to- mesenchymal transition (EMT/ EndMT), extracellular matrix (ECM) remodeling, and related signaling pathways. Overall, DPP-targeted interventions are promising in pulmonary medicine, but broader clinical translation will require well-designed prospective trials.

## 1. Introduction

Dipeptidyl peptidase (DPP) family enzymes are increasingly recognized as multifunctional regulators of inflammation, metabolism, cardiovascular homeostasis, and host defense [1,2]. Among them, DPP-4 has been studied most extensively because of its established role in type 2 diabetes mellitus and the widespread clinical use of DPP-4 inhibitors (DPP-4is) [2,3,4]. Their broad biologic activity has also prompted interest in potential respiratory applications, including during the coronavirus disease 2019 (COVID-19) pandemic [5].

Bronchiectasis remains a burdensome respiratory disease with major effects on health-related quality of life (HRQoL) [6]. Until recently, treatment relied mainly on airway clearance, antibiotics, mucolytics, and pulmonary rehabilitation [6]. The introduction of brensocatib, a reversible DPP-1 (cathepsin C) inhibitor, has reshaped the therapeutic landscape of non-cystic fibrosis bronchiectasis [7]. At the same time, emerging evidence suggests that DPP family members may also contribute to pulmonary hypertension, interstitial lung disease, acute lung injury, asthma, and lung cancer, thereby broadening the clinical relevance of this enzyme family.

The aim of this narrative review is to summarize current basic, translational, and clinical evidence on the role of selected DPP family members and the therapeutic potential of their inhibition in respiratory diseases.

## 2. Literature Search and Selection Criteria

This narrative review integrates available preclinical and clinical evidence on the role of DPP family members in pulmonary diseases. Scopus, PubMed/MEDLINE, and Google Scholar were searched without date restriction using combinations of the terms “dipeptidyl peptidase”, “lung”, and “respiratory system”. Clinical, observational, translational, and experimental studies published in English were considered. Because this is a narrative review, PROSPERO registration was not required.

## 3. Results

### 3.1. The Biological Role of DPP Family

DPPs comprise largely prolyl-specific peptidases/proteases [1]. In mammalian species, the DPP family includes DPP-1 (cathepsin C [CatC, CTSC]), DPP-2 (DPP-7), DPP-3, DPP-4 (cluster of differentiation [CD]26), DPP-6, DPP-8, DPP-9, and DPP-10, whereas DPP-5 and DPP-11 are found only in bacteria [1,8]. A subclassification, the DPP-4 family, comprises DPP-4, DPP-8, DPP-9, fibroblast activation protein (FAP), and the non-enzymes DPP-6 and DPP-10 [9]. DPP-1 is a lysosomal cysteine protease involved in the activation of proinflammatory mediators, including neutrophil serine proteases (NSPs) such as neutrophil elastase (NE), cathepsin G (CatG), proteinase 3 (PR3), and neutrophil serine protease 4 (NSP4) [10]. DPP-2 promotes lymphocyte quiescence and affects interleukin (IL)-17 production [11]; it has also been studied for diagnostic and therapeutic implications in cancer, particularly through effects on macrophages in the tumor microenvironment [12,13,14,15,16]. The physiologic relevance of DPP-3, a zinc-dependent aminopeptidase, is increasingly recognized in blood pressure regulation, inflammation, pain signaling, and cancer [17]. It has also been proposed as a biomarker of increased renin–angiotensin–aldosterone system (RAAS) activity in worsening heart failure, while procizumab has been explored as a targeted therapeutic agent [18,19,20,21]. DPP-4 is the best-studied family member and an established therapeutic target in type 2 diabetes mellitus, with additional possible roles in atherosclerosis [22,23,24]. DPP-6 and DPP-10 modulate Kv4-mediated A-type potassium channels and are considered potential therapeutic targets, particularly in neurodegenerative disorders such as Alzheimer’s disease [25,26,27,28,29]. In addition, DPP-6 methylation has shown promise as a cancer biomarker [30,31]. DPP-8 and DPP-9 are closely related intracellular serine peptidases whose inhibition activates inflammasomes such as caspase recruitment domain-containing protein 8 (CARD8) [32,33,34,35]; both have also been implicated in tumorigenesis, inflammation, and fibrosis, and are being investigated as therapeutic targets, including through repurposing of DPP-4 inhibitors such as vildagliptin [36,37]. Overall, the functions of many DPP family members remain incompletely understood. Although DPP inhibition has clear therapeutic relevance in some conditions, especially type 2 diabetes mellitus, translation into broader clinical practice remains limited, and their role in pulmonary diseases is even less well explored.

### 3.2. The Role of DPPs in Respiratory Diseases

#### 3.2.1. DPP-1

The lysosomal cysteine protease DPP-1 participates in innate immunity, antimicrobial defense, and neutrophilic inflammation [38]. Because bronchiectasis is characterized by persistent neutrophil-driven airway injury, DPP-1 inhibition has emerged as an attractive strategy to reduce activation of multiple NSPs rather than NE alone [39]. DPP-1 inhibition has also been explored in asthma, where it may influence airway remodeling and epithelial dysfunction.

In 2018, AZD7986 (brensocatib) was evaluated in healthy volunteers to characterize its pharmacokinetics, pharmacodynamics, and safety [40]. Across dose-escalation cohorts, the compound reduced NE activity in a dose-dependent manner without lowering neutrophil counts and reached steady effects after a delayed transit period consistent with neutrophil maturation kinetics [40]. Overall tolerability was acceptable, with mainly mild skin-related adverse events reported at higher exposure levels [40].

These findings supported subsequent phase 3 evaluation in 1721 adolescents and adults with non-cystic fibrosis bronchiectasis [41]. Brensocatib significantly reduced the annual rate of pulmonary exacerbations, prolonged time to first exacerbation, and attenuated loss of forced expiratory volume in the first second (FEV1) compared with placebo, while hyperkeratosis remained the most frequent adverse event [41].

Recently, another trial evaluated the underlying molecular mechanisms mediating the therapeutic potential of brensocatib in subjects with bronchiectasis [42]. In this phase 2, double-blind, placebo-controlled trial, termed the WILLOW Trial, the anti-inflammatory properties of brensocatib were investigated in 215 study participants with available sputum samples [42]. Again, subjects were randomized to receive either 10 mg or 25 mg brensocatib or placebo [42]. Treatment with brensocatib significantly boosted the levels of two major antimicrobial peptides, secretory leukoproteinase inhibitor (SLPI) and α-defensin-3 [42]. This effect was observed both at week 4 and at week 24 [42]. For subjects with elevated NE levels, decreased concentrations of mucin (MUC)-5AC were noted, without any changes in myeloperoxidase (MPO) [42]. Several cytokines were increased, such as C-X-C motif chemokine ligand (CXCL)10, C-C motif chemokine ligand (CCL)8, CCL7, CCL3 and IL-6 both at week 4 and at week 28 [42]. In a subgroup of the study, the EMBARC-BRIDGE cohort, NE correlated inversely with SLPI, CCL13, IL-7, CCL11, CXCL10, CCL8, CCL7 and all these markers were upregulated by brensocatib [42].

Another randomized, double-blind, placebo-controlled, parallel-group trial in subjects with non-cystic fibrosis bronchiectasis evaluated the effect of brensocatib-based treatment in 256 study participants with available sputum samples [43]. In line with previous outcomes, NE levels were reduced in a dose-dependent manner, as higher reduction was found with 25 mg brensocatib [43]. Specifically, NE levels were 141 ng/mL with 25 mg brensocatib, 214 ng/mL with 10 mg brensocatib, and 1514 ng/mL with placebo [43]. The effects of brensocatib after its discontinuation were not sustained, as a rise in NE levels was documented 4 weeks after its withdrawal [43].

Brensocatib treatment resulted in a decrease in PR3 activity, but to a lesser extent than for NE and CatG, in a dose dependent response [43]. Specifically, PR3 levels were assessed at 1368 ng/mL with 25 mg brensocatib, 2309 ng/mL with 10 mg brensocatib, and 2927 ng/mL with placebo [43]. White blood cells (WBCs)-specific NE concentrations were assessed at 5.77 ng/mL with placebo, 4.59 ng/mL with 10 mg brensocatib, and 2.17 ng/mL with 25 mg brensocatib [43]. Statistical significance was observed only for the dose of 25 mg (*p* < 0.05) [43]. NE levels were reduced by 20% and 62% for 10 mg and 25 mg brensocatib [43]. The correlation between NE and CatG activity was the strongest (r = 0.81), followed by NE and PR3 (r = 0.62) and PR3 and CatG (r = 0.59) [43]. Notably, NE activity was decreased in sputum to a greater extent than in WBCs [43].

A posthoc analysis of the phase 2 WILLOW trial further suggested that brensocatib prolonged time to first exacerbation across clinically relevant subgroups and attenuated lung-function decline, particularly in milder disease, without new safety signals [44].

Preclinical studies demonstrated a central role for DPP-1 in airway epithelial dysfunction and airway remodeling [45]. In a 7-week house dust mite (HDM)-induced asthma mouse model, as well as in a severe asthma model, increased DPP-1 expression was observed in lung tissue and airway epithelial cells and was associated with basement membrane thickening, goblet cell hyperplasia, and enhanced collagen deposition [45]. Administration of DPP-1 to wild-type (WT) mice induced mucus production and collagen deposition independent of baseline inflammation, whereas *DPP-1* knockout (KO) mice exhibited attenuated remodeling responses [45]. At the cellular level, DPP-1 deficiency resulted in increased epithelial proliferation and E-cadherin expression, alongside reduced vimentin and alpha smooth muscle actin (α-SMA) expression, indicating diminished activation of epithelial–mesenchymal trophic units (EMTUs) [45]. In vitro, DPP-1 overexpression in human bronchial epithelial cells impaired proliferation, barrier integrity, antioxidative capacity, and wound repair [45]. The expression of adhesion molecules such as E-cadherin and zona-occludens 1 (ZO-1) was reduced and human lung fibroblasts (HLF)-1 levels were increased [45]. Mechanistically, DPP-1-induced HLF-1 activation and collagen synthesis were attenuated by pharmacological inhibition of the p38 pathway, whereas extracellular signal-regulated kinase (ERK) and c-Jun N-terminal kinase (JNK) signaling were minimally affected [45].

These mechanistic findings were supported by clinical observations in human subjects, including 21 healthy individuals, 33 patients with mild-to-moderate asthma, and six patients with severe asthma [45]. Gene expression analyses from Gene Expression Omnibus (GEO) datasets and clinical samples revealed progressive upregulation of DPP-1 with increasing asthma severity, with the highest levels observed in severe or uncontrolled asthma [45]. DPP-1 expression negatively correlated with lung function parameters, including FEV1% predicted and FEV1/forced vital capacity (FVC) ratio, indicating an association with airflow limitation [45]. Owing to its strong discriminatory capacity between disease severities (area under the curve [AUC] = 0.98), DPP-1 emerged as a promising biomarker of asthma severity, thereby linking preclinical evidence of DPP-1-driven airway remodeling to clinically relevant functional impairment [45].

Verducatib (BI 1291583) is a novel selective inhibitor of DPP-1, especially designed for the treatment of bronchiectasis [46]. A phase 1 study comprehensively evaluated the pharmacologic profile of BI 1291583 in 54 subjects by focusing on safety, tolerability, pharmacodynamics, and pharmacokinetics [47]. Maximum DPP-1 levels reached 96% with 30 mg in line with the suggested maximum concentration (Cmax) at 30 mg [47]. Further inhibition in DPP-1 was not achieved at a dose of 40 mg BI 1291583 and this finding was in line with the Cmax being assessed at 30 mg [47]. Researchers attempted to quantify the exact effect of every single dose on PR3 levels; decreases by 30%, 20%, 36%, 58%, and 65% were found with 1, 2.5, 5, and 10 mg, respectively [47]. Differences between fed and fasting state were not particularly meaningful and only a delay by 1 h was found in reaching the Cmax under fasting state [7]. Caution is required when co-administering BI 1291583 with itraconazole, as increases by 58% in Cmax and by 116% in AUC0–tz were observed [47]. BI 1291583 emerged a safe therapeutic option and was well-tolerated; in the multiple-dose studies, skin exfoliation was the most common adverse event reported similar to brensocatib [47].

The phase 2 randomized, double-blind, placebo-controlled, dose-finding study (AIRLEAF) aimed to determine the effective dose [48]. A total of 322 subjects with available sputum samples were randomly allocated to 1 mg, 2.5 mg, or 5 mg BI 1291583, or placebo [48]. Time-to-first-exacerbation analysis revealed a dose-dependent benefit for BI 1291583 compared with the control arm (*p* = 0.0448) [48]. Treatment with BI 1291583 at 5 mg, 2.5 mg, and 1 mg was associated with a decreased risk of exacerbation (hazard ratio (HR) [95% confidence interval, CI] 0.71 [0.48 to 1.05], 0.66 [0.40 to 1.08], and 0.93 [0.60 to 1.45], respectively), although these differences were not statistically significant (*p* > 0.05) [48]. The 2.5 mg dose of BI 1291583 produced the greatest FEV1 at week 24 (adjusted mean versus placebo: 52.4 mL, 95% CI −13.8 to 118.6 mL) and at week 48 (47.7 mL, 95% CI −54.9 to 150.4 mL) [48]. Similar findings were reported for FVC: the 2.5 mg dose produced the greatest FVC change at week 24 (adjusted mean versus placebo 80.1 mL, 95% CI −4.9 to 165.1 mL), week 36 (116.7 mL, 95% CI 29.9 to 203.6 mL), and week 48 (132.0 mL, 95% CI −7.6 to 271.6 mL) [48].

These observations suggest that DPP-1 contributes to asthma not only through inflammatory amplification, but also through structural airway remodeling. In particular, the effects on epithelial integrity, epithelial–mesenchymal trophic unit (EMTU) activation, fibroblast activation, and p38-dependent collagen synthesis indicate that DPP-1 may promote mucus hypersecretion and subepithelial remodeling through defined molecular pathways.

#### 3.2.2. DPP-4

Several studies suggest that DPP-4 inhibition can attenuate endothelial inflammation, remodeling, and mesenchymal transition, supporting a possible role in pulmonary hypertension, acute lung injury (ALI), pulmonary fibrosis, asthma, and lung cancer.

##### Pulmonary Hypertension

In a study featuring *DPP-4*KO and WT mice exposed to hypoxic conditions, the potential use of DPP-4 in pulmonary hypertension with chronic hypoxia was investigated [49]. *DPP-4*KO mice had an increased risk of developing severe pulmonary hypertension and increased medial wall thickness compared with control mice, showing that DPP-4 counteracts hypoxia-induced pulmonary hypertension [49]. After *DPP-4*KO following small interfering ribonucleic acid (siRNA) treatment, an upregulation of *TGFB2*, *TGFB3*, and *TGFA* was observed in the subsequent transcriptome analysis of human lung fibroblasts exposed to hypoxic conditions following [49]. Based on these outcomes, DPP-4 suppresses transforming growth factor (TGF)-β signal-regulated fibroblast activation under concurring hypoxic conditions [49].

In complementary bleomycin-based experiments, *DPP-4*KO attenuated right ventricular pressure overload, vascular muscularization, and smooth-muscle-cell proliferation, supporting a role for DPP-4 in TGF-β-driven pulmonary vascular remodeling [50].

Xu et al. [51] comprehensively assessed the therapeutic potential of sitagliptin in pulmonary hypertension using male Wistar rats together with multiple relevant cell culture models, including A549 human alveolar epithelial cells, 16HBE human bronchial epithelial cells (HBECs), human lung fibroblasts, human pulmonary arterial endothelial cells (HPAECs), human pulmonary artery smooth muscle cells (hPASMCs), and U937 human monocytic cells. DPP-4 expression was detected in airway and alveolar epithelial cells, vascular endothelial and smooth muscle cells, as well as inflammatory cells in lung tissue, and was consistently observed across all cell types studied [51]. In vivo, sitagliptin treatment significantly reduced right ventricular systolic pressure (RVSP), pulmonary arterial wall thickness, and the proportion of fully muscularized pulmonary vessels, while also limiting weight gain [51]. At both administered doses (60 and 80 mg/kg), sitagliptin markedly attenuated pulmonary arterial adventitial fibrosis and reduced expression of remodeling-associated markers, including the mesenchymal markers α-SMA, vimentin, and fibronectin, as well as the endothelial markers von Willebrand factor (vWF) and vascular endothelial cadherin (VE-cadherin) [51]. Similar protective effects were observed in both bleomycin- and chronic hypoxia-induced pulmonary hypertension models, where sitagliptin at 80 mg/kg decreased RVSP, pulmonary arterial wall thickness, right ventricular hypertrophy index, and the cross-sectional area of right ventricular cardiomyocytes over a 28-day period [51]. Mechanistic studies indicated that sitagliptin directly modulates pathological hPASMCs activation. Platelet-derived growth factor-BB (PDGF-BB) stimulation markedly enhanced hPASMCs proliferation and migratory capacity, effects that were significantly attenuated by sitagliptin treatment [51]. At the molecular level, sitagliptin increased expression of phosphatase and tensin homolog (PTEN) and suppressed PDGF-BB-induced phosphorylation of protein kinase B (AKT), p38, and ERK1/2, while leaving JNK signaling largely unaffected [51]. Functionally, sitagliptin effectively inhibited PDGF-BB-induced hPASMCs migration in trans well assays, supporting a role for DPP-4 inhibition in limiting vascular remodeling through regulation of hPASMCs signaling and motility [51].

Linagliptin treatment was evaluated in a mouse model of systemic sclerosis-associated pulmonary hypertension [52]. The model was established following bleomycin administration [52]. Improved histopathological features and weight status were found in linagliptin-treated mice [52]. Lung-specific changes in oxidative stress and inflammatory markers were attenuated following linagliptin administration [52]. Specifically, superoxide dismutase (SOD) was enhanced and malondialdehyde (MDA), tumor necrosis factor (TNF)-α and IL-6 levels were decreased [52]. Bleomycin-induced endothelial-to-mesenchymal transition (EndMT) was ameliorated with linagliptin both in vivo and in vitro [52]. Furthermore, linagliptin administration was associated with decreased migration capacity of endothelial cells initially promoted with bleomycin treatment [52]. Concomitant exposure to bleomycin and rapamycin showed that linagliptin-mediated effects may be attributed to the AKT/mammalian target of rapamycin pathway (mTOR) [52].

Monocrotaline-induced pulmonary hypertension was examined in Sprague–Dawley rats [53]. In rats exposed to monocrotaline, elevated glucagon-like peptide-1 receptor (GLP-1R) expression was observed in contrast with almost nonexistent GLP-1R expression in the control arm [53]. Sitagliptin administration suppressed monocrotaline-induced increased GLP-1R expression [53]. Exendin-3, serving as a GLP-1R antagonist reversed the protective role of sitagliptin in the monocrotaline-induced increased RVSP, hypertrophy of pulmonary vascular medial layer, hypertrophy of RV and hypertrophy of cardiomyocytes [53]. Consequently, these results suggest that GLP-1R antagonism could abolish the protective effects of DPP-4is on pulmonary hypertension [53]. Furthermore, the researchers focused on providing evidence regarding the utility of GLP-1R agonism and therefore administered liraglutide, a glucagon-like peptide-1 receptor agonist (GLP-1RA) [53]. Liraglutide administration alleviated monocrotaline-induced increases in RVSP, pulmonary vascular resistance, pulmonary vascular fibrosis, hypertrophy of RV and hypertrophy of cardiomyocytes [53]. Liraglutide administration reduced the presence of CD68+ macrophages around pulmonary arteries and toluidine blue (TB) staining positive mast cells [53]. Liraglutide treatment resulted in amelioration of increased messenger ribonucleic acid (mRNA) levels of TGF-β1, IL-1β, IL-6 and TNF-α in lung tissue [53]. The effect on IL-6 was however only partial [53]. Significantly decreased fibronectin, a mesenchymal marker, and increased VE-cadherin, an endothelial marker, were found with liraglutide [53]. In bleomycin and chronic hypoxia-induced pulmonary hypertension model, liraglutide treatment attenuated increased RVSP, hypertrophy of right ventricle, thickening of pulmonary vascular medial layer, and accumulation of CD68+ macrophages [53]. Elevated DPP-4 and GLP-1 levels were also decreased [53]. In cultured human umbilical vein endothelial cells (HUVECs) exposed to TGF-β1 and IL-1β, EndMT of HUVECs was reduced in a dose-dependent manner [53]. VE-cadherin between neighboring HUVECs was almost non-existent, while mesenchymal marker vimentin increased [53]. Liraglutide treatment resulted in a dose-dependent decrease in vimentin-positive cells undergoing epithelial-to-mesenchymal transition (EMT) [53]. Liraglutide administration resulted in significantly increased VE-cadherin and ZO-1 and decreased α-SMA and vimentin levels [53]. Significantly suppressed phosphorylations of mothers against decapentaplegic homolog (Smad)2/3 and ERK1/2 in a dose-dependent way were found with liraglutide [53]. Exendin-3 administration significantly attenuated the actions of liraglutide in HUVECs expressing vimentin [53].

Overall, the reported effects in pulmonary hypertension converge on several shared mechanistic axes, including modulation of TGF-β-driven fibroblast and smooth muscle cell activation, inhibition of EndMT, and suppression of AKT/mTOR, p38, ERK1/2, and Smad2/3 signaling. The additional implication of GLP-1R-dependent signaling further suggests that DPP-4 inhibition may attenuate pulmonary vascular remodeling through both direct vascular effects and indirect anti-inflammatory mechanisms.

##### LPS-Induced Lung Injury

Interest in DPP-4 has also expanded to lipopolysaccharide (LPS)-induced lung injury. One recent study identified micro ribonucleic acid(miR)-23b-3p as a negative regulator of DPP-4 in human pulmonary microvascular endothelial cells and showed that both miR-23b-3p and sitagliptin attenuated LPS-induced EndMT and fibrosis-related changes, with the strongest effect seen during combined treatment [54].

In LPS-induced human pulmonary microvascular endothelial cells (HPMECs), DPP-4 knockdown resulted in suppressed chemokine release and hyperpermeability in LPS-exposed HPMECs [55]. LPS-induced neutrophil–endothelial adhesion, polymorphonuclear neutrophil (PMN) chemotaxis and trans-endothelial migration were suppressed through DPP-4 knockdown in a combined culture of HPMECs and human PMNs [55]. This was evidenced by decreased AKT, inhibitor of nuclear factor kappa-B kinase subunit (IKK)-α and nuclear factor kappa-light-chain-enhancer of activated B cells (NF-κB) p65 involved in toll-like receptor (TLR)4/NF-κB pathway activation [55]. Immunoprecipitation and liquid chromatography–tandem mass spectrometry showed promising results for exogenous DPP-4 treatment through increased integrin-α5β1 downstream focal adhesion kinase (FAK)/AKT/NF-κB signaling followed by intercellular adhesion molecule (ICAM)-1 upregulation, thus attenuating endothelial inflammation [55].

Vildagliptin was subsequently tested in LPS-exposed mice and attenuated histologic inflammation, fibrosis, and pulmonary vascular EndMT [56]. Comparable protective effects of vildagliptin and linagliptin were also observed in cell-based systems, even in the absence of immune cells or GLP-1 signaling [56].

Sitagliptin also improved experimental LPS-induced lung injury by reducing DPP-4 activity, bronchoalveolar lavage protein and cell counts, proinflammatory cytokines, and histopathologic injury [57]. In vitro, sitagliptin suppressed cytokine release and proliferation in human lung microvascular endothelial cells (HLMVECs), although its effects on barrier permeability were limited in some epithelial and endothelial models [57].

In acute lung injury (ALI), male C57BL/6 mice and pulmonary microvascular endothelial cells (PMVECs) from Sprague–Dawley rats were investigated [58]. The Cell Counting Kit-8 (CCK8) cytotoxicity assay showed no cytotoxic effect for linagliptin in PMVECs [58]. Furthermore, decrease in trans-endothelial electrical resistance (TEER) and fluorescein isothiocyanate (FITC)-dextran leakage [58]. Decreases in VE-cadherin, β-catenin, and ZO-1 levels were attenuated and the maximum effect in intercellular junction proteins was achieved at a dose of 10 μM [58]. Notably, VE-cadherin, β-catenin, and ZO-1 between adjacent PMVECs were restored with linagliptin [58]. Subsequent transmission electron microscopy (TEM) assessment confirmed the protective effect on intercellular junctions [58]. In endothelial cells, similar outcomes along with decreased Evans Blue (EB) extravasation and lung wet/dry ratio were found in lung tissues with linagliptin, further substantiating the protective effect on pulmonary vascular endothelial barrier function in LPS-induced ALI mice [58]. Linagliptin treatment resulted in restoration of LPS-induced lung tissue damage and in decreased levels of MPO and MDA along with increased levels of SOD, pointing to ameliorating effects on interstitial thickening, interstitial hemorrhage, alveolar collapse, and inflammatory infiltration [58]. LPS-induced mitochondrial ROS production was suppressed with linagliptin [58].

In a related ALI model, linagliptin preserved intercellular junction proteins, reduced endothelial leakage, oxidative stress, and lung edema, and improved histologic injury scores in mice and pulmonary microvascular endothelial cells [58].

Mechanistic work linked these effects with restoration of Epac1/AKT signaling, stabilization of VE-cadherin, β-catenin, and ZO-1, and suppression of mitochondrial reactive oxygen species. Pharmacologic inhibition of Exchange Protein Directly Activated by Cyclic adenosine monophosphate (cAMP) 1 (Epac1) or AKT blunted the protective effects of linagliptin, supporting this pathway as a key mediator of endothelial barrier preservation [58].

*DPP-4*KO has also been studied in acute respiratory distress syndrome ARDS models [59]. Although KO animals showed reduced inflammatory cell infiltration and lower levels of several inflammatory mediators, indices of alveolar-capillary barrier function [59].

Inflammatory cell infiltration was reduced, and marked thickening of the alveolar walls was present in KO mice [59]. TNF-α and IL-6 mRNA levels were decreased in alveolar macrophages of KO mice [59]. This was confirmed in cultured mouse lung microvascular endothelial cells, in which increased IL-6 and ICAM-1 levels were found following treatment with DPP-4 siRNA [59]. PECAM-1 expression (CD31), a major endothelial cell-binding protein, was decreased following treatment with DPP-4 siRNA [59].

Collectively, these studies indicate that the protective effects of DPP-4 inhibition in LPS-induced lung injury are mediated mainly through preservation of endothelial barrier integrity and attenuation of inflammatory signaling. Mechanistically, the available data implicate suppression of EndMT, inhibition of AKT/NF-κB-related inflammatory activation, and restoration of Epac1/AKT-dependent intercellular junction stability, although the dysfunction was not consistently improved, indicating that anti-inflammatory effects may be more robust than permeability-related benefits in this setting [59].

##### Pulmonary Fibrosis

Fibrotic lung diseases remain associated with poor outcomes. In lung fibroblasts, sitagliptin counteracted TGF-β-mediated activation, reduced collagen-1, collagen-3, and fibronectin expression, and suppressed Smad3 phosphorylation, supporting a direct anti-fibrotic effect [60].

EMT represents a second potential target of DPP-4 inhibition. In a cell-based study using 16HBE human bronchial epithelial cells, TGF-β1 induced the expected decrease in E-cadherin and increase in α-SMA, whereas DPP-4 inhibition reversed these changes; similar findings in IL-17-stimulated conditions suggested a synergistic profibrotic role for inflammatory signaling and DPP-4 [61].

##### Lung Cancer

Anagliptin has been evaluated in a non-small cell lung cancer (NSCLC) model either alone or together with anti-Programmed Death-Ligand 1 (PD-L1) therapy [62]. Combination treatment reduced tumor-associated macrophages (TAMs) and M2 polarization, increased intratumoral CD8^+^ T cells, and improved antitumor immune activity, while mechanistic work implicated ERK signaling and reduced nicotinamide adenine dinucleotide phosphate oxidase (NOX)1/NOX2-driven oxidative signaling in monocyte/macrophage reprogramming [62].

##### Asthma

The role of DPP-4 in asthma remains less extensively studied. In an ovalbumin-induced model, DPP-4 promoted T helper 17 cell (Th17) polarization, airway inflammation, mucus hypersecretion, goblet-cell hyperplasia, collagen deposition, and EMT, whereas DPP-4 inhibition attenuated these changes [63]. In the same model, alanyl-glutamine further reduced bronchial inflammation, fibrosis, and the Th17/T regulatory cell (Treg) imbalance in the presence of elevated DPP-4 [64]. Mechanistically, alanyl-glutamine increased sirtuin 1 (SIRT1) expression and restrained DPP-4-associated Th17 differentiation; administration of the SIRT1 inhibitor EX527 reversed these benefits and enhanced Smad2/3 phosphorylation [64].

#### 3.2.3. DPP-9

DPP-9 along with DPP-8 interact with nucleotide-binding oligomerization domainproteins-like-receptor containing a pyrin domain 1 (NLRP1) and induce cytokine maturation and caspase-1 activation [65]. DPP-9 serves as a checkpoint regulator of NLRP1 inflammasome through directly interacting with the inflammatory C-terminal fragment of NLRP1 [65]. The role of DPP-9 in the respiratory system has been investigated in a single combined clinical and experimental study [66]. DPP-9 levels were significantly reduced in tumor tissues compared with non-tumor tissues [66]. Among the various NSCLC cell lines studied, the highest DPP-9 levels were found in the A549 cell line, followed by the H1299, H1975 and H1650 cell lines at both protein and at mRNA level [66]. Following transfection of A549 cell with siRNA targeting human DPP-9 (short hairpin ribonucleic acid [shRNA]-1, shRNA-2, shRNA-3 and shRNA-4), reduced DPP-9 expression was achieved [66]. DPP-9 staining was localized in both cell membrane and cytoplasm [66]. DPP-9 staining was increased in squamous cell lung carcinoma and adenocarcinoma tissues [66].

In the clinical component of the only combined clinical and experimental DPP-9 study, elevated DPP-9 expression was associated with lymph-node metastasis, advanced TNM stage, and worse 5-year overall survival (OS), and remained an independent prognostic factor in multivariable analysis [66].

Experimentally, DPP-9 knockdown reduced NSCLC-cell proliferation, migration, invasiveness, and in vivo tumor growth, whereas DPP-8 levels were largely unchanged [66]. These effects were accompanied by shifts in EMT- and apoptosis-related markers, supporting a functional role for DPP-9 in tumor persistence and metastatic behavior [66].

Western blot analysis of tumor xenograft tissues showed elevated expression levels of E-cadherin, MUC1, p53, BAX and apoptotic protease activating factor 1 (APAF1) and decreased expression levels of vimentin and S100A4 in tumors with DPP-9 silencing [66]. In the BALB/c athymic nude mice with DPP-9 silencing, tumor growth was reduced in vivo and similar outcomes were reflected in growth curves [66].

#### 3.2.4. DPP-10

DPP-10 is an inactive peptidase involved in voltage-gated potassium-channel regulation and has increasingly been linked with aspirin/nonsteroidal anti-inflammatory drug (NSAID)-exacerbated respiratory disease [67,68,69]. In a combined clinical and experimental study, higher serum DPP-10 levels were associated with higher TGF-β1 levels and lower FEV1, while anti-DPP-10 treatment in mice reduced ERK phosphorylation and extracellular matrix deposition (ECM) [68].

A separate case–control study further supported a role for DPP-10 in aspirin-exacerbated respiratory disease by linking the rs17048175 polymorphism and increased circulating DPP-10 levels to this phenotype, but not to aspirin-tolerant asthma [69].

Detailed clinical studies of DPP-targeted interventions in pulmonary diseases are summarized in Supplementary Table S1.

## 4. Discussion

The current review summarizes an expanding body of evidence supporting DPP family members as relevant therapeutic targets in pulmonary diseases. Clinical translation is most advanced for DPP-1 inhibition in bronchiectasis, whereas evidence for DPP-4, DPP-9, and DPP-10 remains predominantly preclinical.

Among DPP-1 inhibitors, brensocatib has shown the clearest clinical benefit, with fewer exacerbations, longer time to first exacerbation, and preservation of lung function in bronchiectasis [41,42,43,44]. Verducatib has produced encouraging phase 1 and phase 2 signals, supporting further development of DPP-1 inhibition in neutrophilic airway disease [47,48].

Verducatib treatment was associated in a similar way with reduced exacerbation risk and elevated FEV1 and FVC [48]. In terms of its mechanism of action, scientists placed emphasis on decreased PR3 levels [48]. Both agents had a reliable safety profile, and major adverse events were restricted to cutaneous manifestations, including lichenoid drug eruption, skin exfoliation, fissures, mild hyperkeratosis in the palm, dry and hyperkeratotic scaly patches [40,47].

Evidence for DPP-9 and DPP-10 is currently limited but biologically informative. DPP-9 appears to influence NSCLC progression through effects on proliferation, invasion, apoptosis, and EMT-related signaling, whereas DPP-10 has been linked mainly with aspirin/NSAID-exacerbated respiratory disease and airway remodeling [66,68,69].

DPP-4 is the most widely studied isoenzyme and its therapeutic properties have been appreciated for years. Experimental data points to therapeutic applications in various respiratory conditions, including pulmonary hypertension, idiopathic pulmonary fibrosis, ALI/ARDS, NSCLC, and asthma. In pulmonary hypertension, DPP-4 promoted, at least partly, endothelial dysfunction and EMT [53,55,56,61]. Increased RVSP, pulmonary arterial wall thickness and ratio of completely muscularized vessels, pulmonary arterial adventitial fibrosis increases, hypertrophy of right ventricle, and hypertrophy of cardiomyocytes were common findings in animal models and cell lines studied [51,53]. TGF-β- emerged as a key mediator facilitating these effects [49,50,53,60,61]. DPP-4is targeted these processes by affecting the ERK1/2 pathway [51,53]. Other mediators strongly involved in the pathogenesis of the condition were: PDGF-BB, which facilitated AKT phosphorylation, p38, IKK-α, NF-κB p65, and Smad2/3 [51,53,55]. The AKT/mTOR pathway, TLR4/NF-κB pathway and integrin-α5β1 downstream FAK/AKT/NF-κB pathway were also explored [52,55]. DPP-4is alleviated EMT, as evidenced by shifts in several markers such as E-Cadherin and/or α-SMA [60]. Epigenetic mediators may potentiate response to DPP-4is, such as miR-23b-3p [54]. The capacity of DPP-4is in attenuating inflammation (e.g., decreased TGF-β1, IL-1β, IL-6 and TNF-α levels) and oxidative stress (e.g., decreased MDA) was also confirmed [52,53]. Basic research evidence suggests that DPP-4 inhibition targets the same therapeutic targets throughout the different conditions evaluated, e.g., TGF-β and Smad-3 protein phosphorylation in idiopathic pulmonary fibrosis [60]. Similarly, in ALI/ARDS the beneficial effects on oxidative stress attenuation (e.g., by targeting MDA and SOD [58]) and major anti-inflammatory properties (e.g., by targeting several key inflammatory mediators, including TNF-α, IL-6, IL-8, CXCL1/KC, CCL2/MCP-1, ICAM-1 and CXCL2/MIP-2 [57,59]) confirmed the promising perspective. In few studies focusing on asthma, alleviation of EMT, release of key cytokines, such as IL-4, IL-5, IL-13 and IL-17 and decreased phosphorylation of Smad2/3 through SIRT1 signaling were observed [63,64]. In NSCLC, the ERK signaling pathway stood in line with previous findings in the forefront of DPP-4 inhibition [62]. Furthermore, beneficial effects on TAMs and M2 polarization attenuation were found, pointing to additional mechanisms of action [62]. These actions potentiated the capacity of PD-L1 inhibition [62]. As liraglutide, one of the most widely used GLP-1RAs yielded promising outcomes in pulmonary hypertension, the importance of the incretin effect mediators needs to be evaluated in respiratory conditions; GLP-1 along with glucose-dependent insulinotropic polypeptide is a substrate for DPP-4 [70]. Currently, such single or double agonists are increasingly investigated for lung-specific actions. Tirzepatide treatment has yielded particularly favorable outcomes in obstructive sleep apnea (OSA) [71]. In large-scale studies, the use of GLP-1RAs, but not of DPP-4is was associated with reduced risk for chronic obstructive pulmonary disease (COPD) exacerbations [72,73]. As clinical studies did not yield promising outcomes with DPP-4is, the use of GLP-1RAs in respiratory conditions may be preferred instead due to their implication in the same pathophysiological pathway.

Available evidence on the importance of DPP-9 and DPP-10 in respiratory conditions is extremely limited [66,68,69]. DPP-9 upregulation was specifically linked with lymph node metastases, thus aggravating TNM stage and 5-year OS [66]. In experimental settings, *DPP-9*KO prevented tumor proliferation and enhanced expression of p53, BAX, and APAF1, also reflected in vivo models [66]. DPP-10 may in future be proven particularly valuable in the management of drug-induced exacerbated lung disease [68,69]. This is a severe condition without any causative treatment available [74]. Serum DPP-10 and TGF-β1 levels were elevated and inversely correlated to FEV1 [68]. This effect was more pronounced in NSAIDs recipients and less pronounced in aspirin users [68]. DPP-10 seemed to promote ERK phosphorylation in airway epithelial cells along with extracellular matrix deposition [68]. Anti-DPP-10 treatment has emerged as a promising approach in mitigating these effects and thus may be considered a promising therapeutic option for the years to come [68]. Another study focused exclusively on aspirin-exacerbated respiratory disease and found that rs17048175 polymorphism is particularly associated with DPP-10-mediated exacerbations in aspirin users [69].

**Figure 1:**
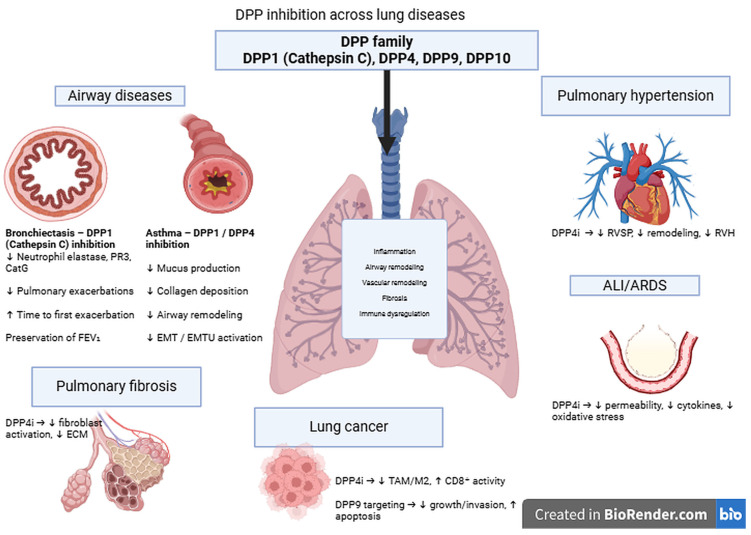
Overview of the role of DPP inhibition in lung diseases. Created in Biorender Drakopanagiotakis, F. (2026) https://BioRender.com/fdrx8ct (accessed on 12 January 2026) (DPP, dipeptidyl peptidase; PR3, proteinase 3; CatG, cathepsin G; EMT, Epithelial to mesenchymal transition; EMTU, epithelial–mesenchymal trophic unit; RVSP, right ventricular systolic pressure; RVH, right ventricular hypertension; ALI, acute lung injury; ARDS, acute respiratory distress syndrome; ECM, extracellular matrix; FEV1, forced expiratory volume in the first second; TAM, tumor-associated macrophage; CD, cluster of differentiation).

This review article encompasses certain advantages, but also considerable limitations. Firstly, it summarizes existing basic research and clinical evidence regarding several DPP-sin several respiratory conditions. Secondly, particular emphasis is placed on underlying molecular mechanisms and their translational importance in clinical practice. However, some DPPs are poorly studied in the respiratory system to date, such as DPP-9 and DPP-10. Scarce, but promising evidence is presented, but its implementation into clinical practice requires further investigation. Other DPPs, such as DPP-2/7 or DPP-3 have not been studied to date in pulmonary conditions. Furthermore, the following contradiction has been observed: the amount of evidence apart from DPP-1 inhibition is almost exclusively based on basic research findings, cell lines or animal models, thus not currently allowing their immediate application in clinical settings despite the wide use of DPP-4is in clinical settings. Overall, large-scale clinical studies are required in order to substantiate further the use of DPP inhibitors in clinical practice. Some progress has been documented for DPP-1. In order to allow clinical translation of the promising findings regarding DPP-4, DPP-9 and DPP-10 into clinical practice, the initiation of clinical trials is necessary. A comprehensive overview of suggested mechanism mediating the beneficial effects of DPP inhibition across the clinical spectrum of lung diseases is given in Table 1 and Figure 1.

## 5. Conclusions

The DPP family has enriched the therapeutic targets in several respiratory conditions. Recently, DPP-1 inhibitors have decisively reshaped the landscape of bronchiectasis pharmacotherapy. Improved lung function and decreased rate of annual exacerbations were major promising results being documented for the affected individuals. DPP-4 inhibitors, as established antidiabetic agents, have yielded promising results in experimental settings for various lung diseases, such as pulmonary hypertension, pulmonary fibrosis, asthma, ALI/ARDS or even lung cancer. Researchers should delineate further which of these conditions is improved with DPP-4is in clinical settings. Scarce, but noteworthy evidence exists to date regarding DPP-9 and DPP-10 inhibition. Due to encouraging evidence, DPP inhibition merits further investigation in future trials.

## Figures and Tables

**Table 1:** Condensed overview of representative DPP-targeted strategies in pulmonary diseases.

DPP Target	Inhibitor(s)	Lung Disease	Representative Evidence	Main Biologic Effects	Principal Mechanisms
DPP-1 (cathepsin C)	Brensocatib, verducatib	Bronchiectasis	Phase 2–3 RCTs; healthy volunteers	↓ exacerbations ↑ time to first exacerbation preserved FEV1 ↓ NE/PR3/CatG	Neutrophil serine protease inhibition
DPP-1 (cathepsin C)	Brensocatib	Asthma	Mouse models; human cohorts	↓ mucus production ↓ collagen deposition ↓ EMTU activation	p38 signaling airway epithelial remodeling
DPP-4/GLP-1R	Sitagliptin, linagliptin, liraglutide	Pulmonary hypertension	Hypoxia, bleomycin, and monocrotaline models	↓ RVSP ↓ vascular remodeling ↓ fibrosis/inflammation	TGF-β/Smad AKT/mTOR ERK1/2 GLP-1R-related signaling
DPP-4	Sitagliptin, linagliptin, vildagliptin	ALI/ARDS	LPS models; endothelial cells	↓ cytokines ↓ permeability/leakage ↓ oxidative stress ↓EndMT	Epac1–AKT NF-κB junction stabilization
DPP-4	Sitagliptin, anagliptin	Pulmonary fibrosis/ NSCLC	Fibroblast, epithelial, and tumor models	↓ fibroblast activation and EMT ↓ M2 polarization ↑ CD8^+^ T-cell activity	TGF-β/Smad ERK signaling macrophage reprogramming
DPP-9	Genetic knockdown	NSCLC	Human tumors, cell lines, xenografts	↓ proliferation ↓ invasion ↓ tumor growth	Apoptosis-related pathways EMT-related modulation
DPP-10	Anti-DPP-10 strategies	NSAID-exacerbated respiratory disease	Human cohorts; murine models	↓ ECM deposition association with better lung function	ERK phosphorylation TGF-β1 signaling

Selected representative studies are summarized to highlight the principal disease settings, therapeutic approaches, major biologic effects, and dominant mechanistic pathways discussed in the review. Detailed clinical study characteristics are provided separately in Supplementary Table S1. (RCTs, randomized controlled trials; CatG, cathepsin G; FEV1, forced expiratory volume in the first second; DPP, dipeptidyl peptidase; PR3, proteinase 3; EMTU, epithelial–mesenchymal trophic unit; RVSP, right ventricular systolic pressure; TGF, transforming growth factor; EndMT, endothelial-to-mesenchymal transition; GLP-1R, glucagon-like peptide-1 receptor; ALI, acute lung injury; ARDS, acute respiratory distress syndrome; LPS, lipopolysaccharide; NSCLC, non-small cell lung cancer; TAMs, tumor-associated macrophages; ECM, extracellular matrix; NSAID, nonsteroidal anti-inflammatory drug).

## Data Availability

No new data were created or analyzed in this study.

## Supplementary Materials

The following supporting information can be downloaded at: https://www.mdpi.com/article/10.3390/biomedicines14051008/s1, Supplementary Table S1: Clinical studies on DPP inhibitors in pulmonary conditions.

## Abbreviations

The following abbreviations are used in this manuscript:

AKT	protein kinase B
ALI	acute lung injury
APAF1	apoptotic protease activating factor 1
ARDS	acute respiratory distress syndrome
AUC	area under the curve
BSI	bronchiectasis severity index
CARD8	caspase recruitment domain-containing protein 8
CatC/CTSC	cathepsin C
CatG	cathepsin g
CCK8	cell counting kit-8
CCL	C-C motif chemokine ligand
CD	cluster of differentiation
CI	confidence interval
Cmax	maximum concentration
COPD	chronic obstructive pulmonary disease
COVID-19	coronavirus disease 2019
CXCL	C-X-C motif chemokine ligand
DPP(s)	dipeptidyl peptidase(s)
DPP-1is	dipeptidyl peptidase-1 inhibitors
DPP-4is	dipeptidyl peptidase-4 inhibitors
EB	Evans Blue
ECM	extracellular matrix
EMT	epithelial-to-mesenchymal transition
EMTUs	epithelial–mesenchymal trophic units
EndMT	endothelial-to-mesenchymal transition
Epac1	exchange protein directly activated by cyclic adenosine monophosphate (cAMP) 1
ERK	extracellular signal-regulated kinase
FAK	focal adhesion kinase
FAP	fibroblast activation protein
FEV1	forced expiratory volume in the first second
FITC	fluorescein isothiocyanate
FVC	forced vital capacity
GEO	Gene Expression Omnibus
GLP-1	glucagon-like peptide-1
GLP-1R	glucagon-like peptide-1 receptor
GLP-1RAs	glucagon-like peptide-1 receptor agonists
HBECs	human bronchial epithelial cell(s)
HDM	house dust mite
HLF	human lung fibroblasts
HLF-1	human lung fibroblasts
HLMVECs	human lung microvascular endothelial cells
HPAECs	human pulmonary arterial endothelial cells
hPASMCs	human pulmonary artery smooth muscle cells
HPMECs	human pulmonary microvascular endothelial cells
HR	hazard ratio
HRQoL	health-related quality of life
HUVECs	human umbilical vein endothelial cells
ICAM	intercellular adhesion molecule
IKK	inhibitor of nuclear factor kappa-B kinase subunit
IL	interleukin
JNK	c-Jun N-terminal kinase
KO	knockout
LLC	Lewis lung carcinoma
LPS	lipopolysaccharide
MDA	malondialdehyde
miR	micro ribonucleic acid
MMP	matrix metalloproteinase
MPO	myeloperoxidase
mRNA	messenger ribonucleic acid
mTOR	mammalian target of rapamycin
MUC	mucin
NE	neutrophil elastase
NF-κB	nuclear factor kappa-light-chain-enhancer of activated B cells
NOX	nicotinamide adenine dinucleotide phosphate oxidase
NSAID(s)	nonsteroidal anti-inflammatory drug(s)
NSCLC	non–small cell lung cancer
NSP(s)	neutrophil serine protease(s)
OS	overall survival
OSA	obstructive sleep apnea
PDGF-BB	platelet-derived growth factor-BB
PD-L1	Programmed Death-Ligand 1
PECAM-1	platelet endothelial cell adhesion molecule
PMN(s)	polymorphonuclear neutrophil(s)
PMVECs	pulmonary microvascular endothelial cells
PR3	proteinase 3
PTEN	phosphatase and tensin homolog
qPCR	quantitative Polymerase Chain Reaction
RAAS	renin–angiotensin–aldosterone system
ROS	reactive oxygen species
RVH	right ventricular hypertension
RVSP	right ventricular systolic pressure
S100A4	S100 calcium-binding protein A4
SE	Standard error
shRNA	short hairpin ribonucleic acid
siRNA	small interfering ribonucleic acid
SIRT1	sirtuin 1
SLPI	secretory leukoproteinase inhibitor
Smad	mothers against decapentaplegic homolog
SNAIL	zinc finger protein SNAI1
SOD	superoxide dismutase
TAM(s)	tumor-associated macrophage (s)
TB	toluidine blue
TEER	trans-endothelial electrical resistance
TEM	transmission electron microscopy
TGF	transforming growth factor
Th17	T-helper 17 cell
TLR	toll-like receptor
tmax	peak plasma concentration
TNF	tumor necrosis factor
Treg	regulatory T cell
VE-cadherin	vascular endothelial cadherin
vWF	von Willebrand factor
WBCs	white blood cells
WT	wild-type
YKL-40	chitinase-3-like protein 1
ZO-1	zona-occludens 1
α-SMA	α-smooth muscle actin
