# Supplementary material for: The Role of Dipeptidyl Peptidase Inhibitors in Pulmonary Diseases
Source: Biomedicines. 2026 Apr 28;14(5):1008. doi: 10.3390/biomedicines14051008 (PMC13203657; doi:10.3390/biomedicines14051008)
Supplement: Supplementary file 1 [file biomedicines-14-01008-s001.zip › biomedicines-4136187-supplementary.pdf]

Supplementary Table 1. Clinical studies on DPP inhibitors in pulmonary conditions. This supplementary table provides a concise study-level overview of the clinical and translational evidence discussed in the manuscript, including trial design, study population, principal findings, and overall clinical relevance.

Study	Study type	Study population & design	Major outcomes	**Conclusions**
Palméret al. (2018) [40]	randomized, placebo-controlled trial	81 healthy individuals treated with Brensocatib or placebo	-Brensocatib maximum plasma levels were found after 0.5 to 1.5 hours and thehalf time of a single dose was found at 20-26 hours<br>-In the 40 mg dose group, NE activity emerged reduced by 50%, without any effect on neutrophil count<br>-The time frame towards reaching steady levels of NE activity was assessed at 25 days<br>-The safety profile was reliable; mild skin adverse events were reported	-Brensocatib was regarded a safe and effective drug
Chalmers et al. (2025) [41]	phase 3, double-blind, randomized controlled trial	1721 subjects (1680 adults and 41 adolescents) treated with brensocatib or placebo	-Significantly reduced annual rate of pulmonary exacerbations was found in groups receiving brensocatib compared with the control arm (rate ratio, brensocatib vs. placebo, 0.79 [95% confidence interval CI, 0.68 to 0.92; adjusted p = 0.004] with the 10 mg dose and 0.81 [95% CI, 0.69 to 0.94; adjusted p = 0.005] with the 25 mg dose)<br>- HR values for the time period to the first exacerbation were in a similar manner significantly decreased: 0.81 (95% CI, 0.70 to 0.95; adjusted p = 0.02) with the 10 mg dose and 0.83 (95% CI, 0.70 to 0.97; adjusted p = 0.04) with the 25 mg dose<br>-Àt week 52, exacerbation-free state was reached for 48.5% of study participants on brensocatib compared with 40.3% in the control arm (rate	-Brensocatib holds therapeutic potential in bronchiectasi s

			ratio, 1.20 [95% CI, 1.06 to 1.37; adjusted p = 0.02] with the 10 mg dose and 1.18 [95% CI, 1.04 to 1.34; adjusted p = 0.04] with the 25 mg dose)<br>-Brensocatib attenuated FEV1 loss, as a reduction by 50 ml with the 10 mg dose, 24 ml with the 25 mg dose and 62 ml with placebo were observed (least-squares mean difference vs. placebo, 11 ml [95% CI, -14 to 37; adjusted p = 0.38] with the 10 mg dose and 38 ml [95% CI, 11 to 65; adjusted p = 0.04] with the 25 mg dose)<br>-Hyperkeratosis was the major side effect reported	
Johnson et al. (2025) [42]	Phase 2, Double-Blind, Placebo-controlled trial (WILLOW Trial)	215 study participants with available sputum samples<br>71 study participants with available sputum samples from 82 study participants randomized to 10 mg brensocatib<br>71 study participants with available sputum samples from 87 study participants randomized to 25 mg brensocatib<br>73 study participants with available sputum samples from 87 study participants randomized to placebo	-The antimicrobial peptides SLPI and α-defensin-3 were significantly increased in subjects on brensocatib at week 4 and at week 24<br>-Reduced levels of mucin-5AC were found, mainly in subjects with elevated NE levels<br>-No changes in MPO levels were found<br>-The levels of 15 cytokines were found upregulated; increased levels of CXCL10, CCL8, CCL7, CCL3 and IL-6 were observed both at week 4 and at week 28	-The anti-inflammatory properties of brensocatib are notable beyond its impact on serine proteases
Cipolla et al. (2023) [43]	randomized, double-blind, placebo-controlled, parallel-group trial in subjects with non-cystic fibrosis bronchiectasis	256 study participants with available sputum samples<br>89 study participants with available sputum	-NE levels were reduced in a dose-dependent way with brensocatib and more pronounced decreases were found with 25 mg compared to 10 mg	-Brensocatib affects decisively proteinase activity

		samples on 25 mg brensocatib<br>81 study participants with available sputum samples on 10 mg brensocatib<br>85 study participants with available sputum samples on placebo	-NE concentrations were assessed at 1514 ng/mL for placebo, 214 ng/mL for 10 mg brensocatib, and 141 ng/mL for 25 mg brensocatib<br>-4 weeks after brensocatib discontinuation, NE levels increased<br>-CatG concentrations PR3 activity was reduced to a lesser extent than for NE and CatG, but also showed a dose-dependent response<br>-PR3 concentrations were assessed at 2927 ng/mL for placebo, 2309 ng/mL for 10 mg brensocatib, and 1368 ng/mL for 25 mg brensocatib<br>-WBCs-specific NE concentrations were assessed were assessed at 2927 ng/mL for 5.77, 4.59 ng/mL for 10 mg brensocatib, and 2.17 ng/mL for 25 mg brensocatib ($p < 0.05$) only for 25 mg); NE levels were reduced by 20% and 62% for 10 mg and 25 mg brensocatib<br>-The correlation between NE and CatG activity was the strongest (r=0.81), followed by NE and PR3 (r=0.62) and PR3 and CatG (r=0.59)	
Chalmers et al. (2025) [44]	Post-hoc analysis of the phase 2 WILLOW trial	256 study participants	-Treatment with brensocatib resulted in a longer time to first exacerbation (HR (95% CI), BSI: ≤4, 0.28 (0.08-0.96); 5-8, 0.75 (0.35-1.60); ≥9, 0.61 (0.35-1.04); prior exacerbations: 2, 0.56 (0.34-0.90); ≥3, 0.71 (0.32-1.59); blood eosinophils per μL: <300, 0.66 (0.42-1.06); ≥300, 0.49 (0.20-1.20); long-term macrolide use: no, 0.60 (0.38-0.94); yes, 0.60 (0.25-1.45); P. aeruginosa culture: negative, 0.54 (0.32-0.92); positive, 0.68 (0.37-1.27)) compared to the control arm<br>-Brensocatib attenuated lung function loss, with a more	-Brensocatib showed benefits in the post-hoc analysis

			pronounced effect on mild disease (mL difference (SE) for patients with BSI ≤4, 95.7 (71.3); two previous exacerbations, 46.7 (33.7); blood eosinophil count<br>-The safety profile was consistent within treatment groups	
Yuan et al. (2024) [45]	Comparative study<br>Combined experimental and clinical study	Combined clinical and experimental study<br>21 healthy individuals<br>33 subjects with mild-to-moderate asthma<br>6 subjects with severe asthma<br>6- to 8-week-old WT C57BL/6 mice (CTSC+/+) or CTSC-knockout mice (CTSC-/-)<br>immortalized HBE cell line 16HBE14o- cells	-In gene expression profiles from GEO, CTSC upregulation was found in severe or uncontrolled asthma compared to healthy controls<br>-CTSC levels were increased in subjects with mild-to-moderate asthma and further increased in subjects with severe asthma compared to healthy individuals<br>-A negative correlation between CTSC levels and FEV1% and FEV1/FVC was established<br>-CTSC levels may also serve as potential biomarker with AUC at 0.98<br>-Chronic administration of CTSC in WT mice resulted in enhanced mucus production and collagen deposition, independent of preexisting airway inflammation<br>-In a seven-week house dust mite-induced asthma model with airway remodeling, mucus production and collagen deposition were attenuated in CTSC-/-mice compared with CTSC+/+ mice after house dust exposure<br>-In a severe asthma model, collagen deposition was enhanced in CTSC-/-mice compared with CTSC+/+ mice, without any difference on mucus production<br>-Increased expression levels of CTSC in the lung and airway epithelial cells was found in	-AZD7986 showed beneficial effects in asthma

			both the house dust mite model and the severe asthma model compared to control<br>-Compared with CTSC+/+ mice, elevated levels of Ki67 and E-cadherin in airway epithelial cells and deceased levels of vimentin and α-SMA<br>-In HBECs with overexpression, decreased HBECs proliferation with increased permeability coefficient of HBECs monolayers, decreased antioxidative activity and diminished levels of cell adhesion molecules, such as E-cadherin and ZO-1 were found<br>-Increased HLF-1 levels were observed following CTSC administration<br>-The administration of p38 inhibitor (SB203580) resulted in reduced activation of HLF-1 and collagen synthesis induced by CTSC<br>-Elevated levels of IL-5, IL-13, and IL-17A and decreased inflammatory cell presence were found in CTSC-/-mice following house dust mite exposure and similar outcomes without an increase in IL-5 and IL-17A levels were observed in the severe asthma model of CTSC-/-mice<br>-AZD7986 administration in mice exposed to house dust mite resulted in decreased lung inflammation, mucus production, collagen deposition and EMTU activation	
Badorrek et al. (2024) [47]	phase I characterization study<br>(a single-rising-dose study (NCT03414008) and two multiple-rising-dose studies	54 subjects in the single-rising-dose study<br>24 subjects in each multiple-rising-dose study	-BI 1291583 emerged a safe therapeutic option and was well-tolerated; in the multiple-dose studies, skin exfoliation was the most common adverse event reported	-BI 1291583 showed benefits and had a reliable safety profile in healthy individuals

	(NCT03868540 and NCT04866160)	12 subjects in the food effect study<br>14 subjects in the drug-drug interaction study	-tmax was reached at 6 hours; t1/2 varied considerably between 33.6 h with 25 mg to 60.2 h in the 40 mg<br>-Maximum CatC reached 96% with 30 mg in line with Cmax at 30 mg<br>-Reduced PR3 levels by 30%, 20%, 36%, 58%, and 65% were found with 1, 2.5, 5, and 10 mg, respectively<br>-Similar Cmax, AUC0-tz and t1/2 were found between fasting and fed state, while the Cmax was reached after 6 under fed and 7 h under fasted states<br>-Itraconazole administration resulted in increased Cmax by 58% and AUC0-tz by 116%, as well as increased t1/2	
Chalmers et al. (2025) [48]	phase 2 randomised, double-blind, placebo-controlled, dose-finding study (AIRLEAF)	322 study participants with available sputum samples<br>53 study participants on 1 mg BI 1291583<br>53 study participants on 2.5 mg BI 1291583<br>107 study participants on 5 mg BI 1291583<br>109 study participants on placebo	-Time to first exacerbation analysis revealed adose-dependent benefit for BI 1291583 compared to treatment with placebo (p = 0.0448)<br>-BI 1291583 treatment at 5 mg 2.5 mg and 1 mg (HR (95% CI) 0.71 (0.48 to 1.05), 0.66 (0.40 to 1.08) and 0.93 (0.60 to 1.45), resulted in decreased risk of exacerbation (p>0.05)<br>-The BI 1291583 2.5 mg dose resulted in the greatest FEV1 at week 24 (adjusted mean versus placebo: 52.4 mL, 95% CI −13.8 to 118.6 mL) and at week 48 (47.7 mL, 95% CI −54.9 to 150.4 mL)<br>- The BI 1291583 2.5 mg dose resulted in the greatest FEV1 change at week 24 (adjusted mean versus placebo: 52.4 mL, 95% CI −13.8 to 118.6 mL) and at week 48 (47.7 mL, 95% CI −54.9 to 150.4 mL)<br>-The BI 1291583 2.5 mg dose resulted in the greatest FVC change at week 24 (adjusted	-The risk of exacerbation in adults with bronchiectasis was reduced following treatment with BI 1291583

			mean versus placebo 80.1 mL, 95% CI −4.9 to 165.1 mL)and at week 36 (116.7 mL, 95% CI 29.9 to 203.6 mL) and at week 48 (132.0 mL, 95% CI −7.6 to 271.6 mL)<br>-The safety profile was similar to the control arm	
Tang et al. (2017) [66]	Combined clinical and experimental study	217 subjects with NSCLC<br>Human NSCLC cell lines A549, NCI-H1299, NCI-H1675 and NCI-H1650<br>Male BALB/c-nude mice	-DPP-9 levels were significantly reduced in tumor tissues compared to non-tumor tissues<br>-Highest DPP-9 levels were found in A549 cell line, followed by NCI-H1299, NCI-H1975 and NCI-H1650 cell lines<br>-Following transfection of A549 cell with RNA targeting human DPP-9 (shRNA-1, shRNA-2, shRNA-3 and shRNA-4), reduced DPP-9 expression; similar outcomes were observed in shDPP-9-transfected NSCLC cells<br>-DPP-9 staining was observed in cell membrane and cytoplasm; DPP-9 staining was increasingly in squamous cell lung carcinoma and adenocarcinoma tissues<br>-Elevated DPP-9 expression was found in 131 (60.37%) study participants, while decreased or absent DPP-9 expression in 86 (39.63%) of study participants; significant correlation between DPP-9 expression and lymph node metastases (p = 0.028) and TNM stage (p = 0.040) could be documented<br>-Survival analysis indicated that DPP-9 overexpression was a negative prognostic factor for 5-year overall survival (HR 1.639, 95% CI 1.210-2.220, p 5 0.001); Cox regression analysis showed that DPP-9 overexpression	-DPP-9 plays a crucialoncogenic role of NSLCL

			was an independent prognostic factor for 5-year OS (HR 1.581, 95% CI 1.164-2.146, p 5 0.003)<br>-DPP-9 knockdown suppressed proliferation ability; cell motility and invasiveness were significantly reduced in shDPP-9-transfected cells, compared with control cells in migration assays and Matrigel invasion assay; DPP8 protein levels were unaffected<br>-In H1650 cells, cell motility in shDPP-9-transfected cells was accelerated, E-cadherin and MUC1 were decreased and vimentin, S100A4 and SNAIL were increased<br>-In shDPP-9-transfected cells, increased expression of p53, BAX, and APAF1 was observed<br>-Western blot analysis of tumor xenograft tissues showed elevated expression levels of E-cadherin, MUC1, p53, BAX and APAF1 and decreased expression levels of vimentin and S100A4 in tumors with DPP9 silencing<br>-In the BALB/c athymic nude mice with DPP-9 silencing, tumor growth was reduced in vivo; similar outcomes were obtained in growth curves	
Sim et al. (2022) [68]	Combined clinical and experimental study	110 subjects with NSAIDs-exacerbated respiratory disease<br>130 subjects with aspirin-tolerant asthma<br>80 healthy individuals<br>6-week-old female and male BALB/c mice	-In subjects with NSAIDs-exacerbated respiratory disease, elevated levels of serum DPP-10 and TGF-β1 with lower FEV1 than aspirin-tolerant asthma subjects or the control arm ($p < 0.05$ for each comparison)<br>-In subjects with NSAIDs-exacerbated respiratory disease and increased DPP-10 levels, higher TGF-β1and	-DPP-10 may contribute to TGF-β1-mediated airway dysfunction in subjects with NSAID-exacerbated respiratory disease

			lower FEV1 were found ($p < 0.05$ for all)<br>-Serum DPP-10 levels had a positive correlation with TGF-β1 ($r=0.384$, $p < 0.001$), but a negative correlation with FEV1 ($r=-0.230$, $p = 0.016$) in subjects with NSAIDs-exacerbated respiratory disease<br>-In vitro studies showed expression of DPP-10 in airway epithelial cells enhanced by TGF-β1 treatments<br>-DPP-10 production was mediated by from immune cells and DPP-10 promoted ERK phosphorylation in airway epithelial cells, which was suppressed by anti-DPP-10 treatment<br>- In asthma mouse models, elevated serum DPP-10 levels and TGF-β1 levels in the BAL were observed, which were suppressed by anti-DPP-10 treatment.<br>- Anti-DPP-10 treatment reversed ERK phosphorylation and extracellular matrix deposition in the lung	
Kim et al. (2015) [69]	Case-control association study	274 subjects with aspirin-exacerbated respiratory disease<br>272 subjects with aspirin-tolerant asthma<br>99 healthy individuals	-A significant association between rs17048175 and the aspirin-exacerbated respiratory disease phenotype was established, but not with aspirin-tolerant asthma<br>-DPP-10 levels were significantly elevated in subjects with aspirin-exacerbated respiratory disease compared to subjects with aspirin-tolerant asthma and control subjects ($p = 0.021$ and $p < 0.001$, respectively)<br>-A significant correlation between serum DPP-10 levels a 15-hydroxyeicosatetraenoic acid ($r=0.226$, $p = 0.017$) and	-The genetic contribution of rs17048175 to DPP-10 in eosinophilic inflammation is crucial in aspirin-exacerbated respiratory disease

			YKL-40 (r=0.364, p = 0.004) was established	

(NE, neutrophil elastase; CI, confidence interval; SLPI, secretory leukoproteinase inhibitor; MPO, myeloperoxidase; CXCL, C-X-C motif chemokine ligand; CCL, chemokine [C-C motif] ligand; IL, interleukin; CatG, cathepsin G; CatC/CTSC, cathepsin C; PR3, proteinase 3; WBC, white blood cell; FEV, forced expiratory volume; FVC, forced vital capacity; BSI, bronchiectasis severity index; GEO, Gene Expression Omnibus; WT, wild type; HBECs, human bronchial epithelial cells; HLF-1, human lung fibroblasts; NSCLC, non-small cell lung cancer; DPP, dipeptidyl peptidase; shRNA, short hairpin ribonucleic acid; HR, hazard ratio; OS, overall survival; SNAIL, zinc finger protein SNAI1; MUC, mucin; S100A4, S100 calcium-binding protein A4; APAF1, apoptotic protease activating factor 1; NSAIDs, nonsteroidal anti-inflammatory drugs; FEV1, forced expiratory volume in 1 second; ERK, extracellular signal-regulated kinase; BAL, bronchoalveolar lavage; YKL-40, chitinase-3-like protein 1 [CHI3L1]; EMTU, epithelial-mesenchymal trophic unit; tmax, peak plasma concentration, Cmax, maximum concentration).
